# Change in Japanese Encephalitis Virus Distribution,Thailand

**DOI:** 10.3201/eid1411.080542

**Published:** 2008-11

**Authors:** Narong Nitatpattana, Audrey Dubot-Pérès, Meriadeg Ar Gouilh, Marc Souris, Philippe Barbazan, Sutee Yoksan, Xavier de Lamballerie, Jean-Paul Gonzalez

**Affiliations:** Mahidol University, Bangkok, Thailand (N. Nitatpattana, A. Dubot-Pérès, M. Ar Gouilh, M. Souris, S. Yoksan, P. Barbazan, J-P. Gonzalez); Institut de Recherche pour le Dévelopement, Paris, France (M. Dubot-Pérès, M. Argouih, P. Barbazan, J-P Gonzalez); Université de la Méditerranée, Marseille, France (X. Lamballerie); International Center for Medical Research of Franceville, Franceville, Gabon (J.-P. Gonzalez)

**Keywords:** Japanese encephalitis virus, genotype, Thailand, dispatch

## Abstract

Japanese encephalitis virus (JEV) genotypes in Thailand were studied in pigs and mosquitoes collected near houses of confirmed human JEV cases in 2003–2005. Twelve JEV strains isolated belonged to genotype I, which shows a switch from genotype III incidence that started during the 1980s.

The origin of Japanese encephalitis virus (JEV) was recognized before 1935, and JEV was isolated in Japan in 1935. The virus has since spread from India to Indonesia and within the past 3 decades has reached previously unaffected parts of Asia and northern Australia (*1*,*2*). JEV is one of the most widespread causes of viral encephalitis worldwide; an estimated 3 billion persons are at risk for infection, and 10,000 to 15,000 die annually (*3*). Although most human infections are asymptomatic (1/1,000), 1/300 infections causes symptomatic infections, and 1/4 patients seeking treatment have symptoms of brain inflammation, which can lead to permanent neurologic sequelae and a 1/4 death rate (*4*).

 JEV is a flavivirus transmitted by *Culex* mosquitoes to birds and pigs; humans are dead-end incidental hosts. On the basis of nucleotide sequencing of capsid/premembrane protein (C/PrM) and envelope (E) genes, 5 virus genotypes have been identified, including genotypes I to III (GI, GII, GIII). These have been found distributed all over southern Asia; a GIV strain was isolated from eastern Indonesia, and an isolate originating in Malaysia may represent a fifth genotype (*5*).

Three vaccines, derived from JEV GIII strains, are currently in use. Since the 1960s JEV immunization campaigns have dramatically reduced the effects of the disease in southern and Southeast Asia (*6*). In Thailand, JEV immunization began as a part of childhood vaccination program in the northern provinces in 1990; this program rapidly expanded to 36 provinces that had reported a persistent incidence of encephalitis (*7*).

## The Study

**Figure 1:**
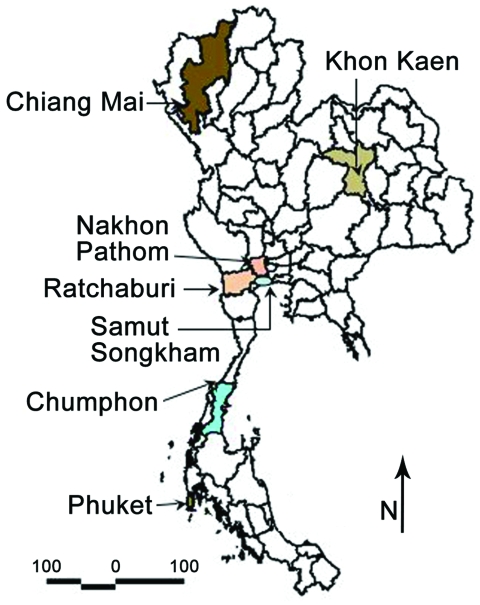
Provinces of Thailand showing study sites in Phuket, Chiang Mai, Ratchaburi, Nakhon Pathom, Khon Kaen, Chumphon, and Samut Songkham.

To study the JEV genotype distribution in Thailand and to eventually detect changes in Japanese encephalitis epidemiologic patterns, we conducted a 3-year survey (2003–2005) of JEV incidence in 7 provinces representative of the 4 regions of Thailand (north, Chiang Mai Province; northeast, Khon Khen Province; central plain, Nakhon Pathom, Ratchaburi, and Samut Songkram Provinces; south, Phuket and Chumphon Provinces). Pig farms and rice fields within a 2-km radius around houses of confirmed human cases of Japanese encephalitis were targeted for sample collection. Ten healthy sentinel piglets (10 weeks of age) were surveyed in each province, and blood samples were collected weekly for 14 weeks. Adult mosquitoes were collected on a monthly basis according to the targeted pig farm and availability of breeding sites for vectors (Table 1; Figure 1) by using both the CDC gravid trap (Model 1712) and the CDC light trap (P. Reiter, Centers for Disease Control and Prevention, Fort Collins, CO, USA).

**Table 1:** Location of Japanese encephalitis virus study sites, Thailand

Site no.	Province	Latitude N	Longitude E
1	Phuket	43º36′90′′	88º67′10′′
2	Chiang Mai	50º10′10′′	21º15′757′′
3	Ratchaburi	58º41′09′′	15º19′015′′
4	Nakhon Pathom	59º38′49′′	15º46′044′′
5	Khon Kaen	18º63′72′′	18º28′276′′
6	Chumphon	09º58′09′′	99º02′87′′
7	Samut Songkham	13º26′24′′	100º00′00′′

**Figure 2:**
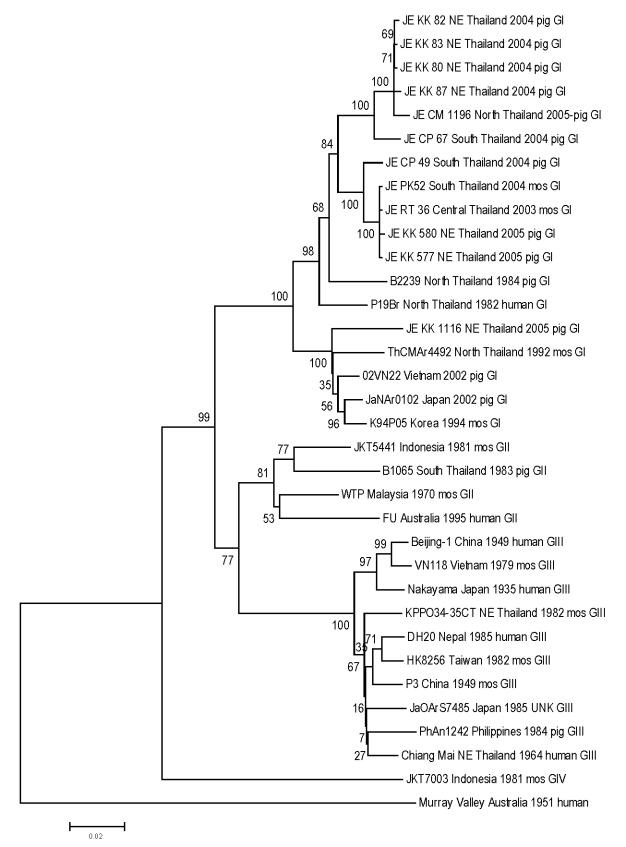
Sequence phylogeny based on E (envelope) gene nucleotide sequence of Japanese encephalitis virus isolates from pigs and mosquito hosts in Thailand during 2003–2005, with reference to other Southeast Asian isolates. Phylogenetic analysis was performed by using nucleotide alignments, the Kimura 2-parameter algorithm (for the calculation of pairwise distances), and the neighbor-joining method (for tree reconstruction), as implemented in MEGA software (*9*). The tree was rooted within the Japanese encephalitis serogoup by using Murray Valley virus (GenBank accession nos. E1–51). The robustness of branching patterns was tested by 1,000 bootstrap pseudoreplications. Each strain is abbreviated, followed by the country of origin (and the region of origin in Thailand, e.g., NE = northeast) and year of isolation. Bootstrap values are indicated above the major branch; 33 taxa comprised the ingroup, and all taxa were rooted with Murray Valley virus. A unique gap was treated as a "fifth base." The character state optimization was chosen as accelerated transformation. Consistence index 0.572; retention index 0.7528.

Fifty microliters each from pig serum specimens and from filtered suspension of crushed mosquitoes were used for virus isolation on C6/36 cells. We tested JEV propagation by immunofluorescent assay. RNA extraction was done from a supernatant of JEV-positive cell culture, after first passage, according to manufacturer’s protocol as well as RNA reverse transcription–PCR (RT-PCR) (GIBCO-BRL, Gaithersburg, MD, USA). RT-PCR was performed on 4 μL of cDNA template by using 2.5 units of AmpliTaq Gold DNA Polymerase (PerkinElmer, Foster City, CA, USA). Overlapping JEV E gene fragments were amplified with 2 sets of primers: Ea forward primer (5′-ATA GTA GCT ATG TGT GCA AAC AAG G 5-3′), Ea reverse primer (5′-GAA TTC RGT YGT GCC YTT CAG AGC-3′); and Eb forward primer (5′-AGC TCA GTR AAG TTR ACA TCA GG-3′), Eb reverse primer (5′-GAA TTC AAT GGC ACA KCC WGT GTC-3′), respectively (*8*). The 1,216 nucleotides generated partial sequences of the JEV E gene that were compiled by using Sequence-Alignment Editor software version 2.0a11 (A. Rambaut, Department of Zoology, University of Oxford, Oxford, UK); pairwise genetic distances were calculated with MEGA software version 2.0 (*9*) (Table 2; Figure 2).

**Table 2:** Strains of Japanese encephalitis virus used for phylogenetic analysis*

Strain	Year	Location	Source	Genotype	GenBank accession no.
FU	1995	Australia	Human	II	AF217620
P3	1949	China	Mosquito†	III	AY243844
Beijing-1	1949	China	Human brain	III	L48961
JKT7003	1981	Indonesia	Mosquito†	IV	U70408
JKT5441	1981	Indonesia	Mosquito†	II	U70406
Nakayama	1935	Japan	Human brain	III	AF112297
JaOArS7485	1985	Japan	Unavailable	III	AB028259
JaNAr0102	2002	Japan	Pig blood	I	AY377577
K94P05	1994	Korea	Mosquito†	I	U34929
WTP	1970	Malaysia	Mosquito†	II	U70421
DH20	1985	Nepal	Human brain	III	U03690
PhAn1242	1984	Philippines	Pig	III	U70417
HK8256	1982	Taiwan	Mosquito†	III	U70396
Chiang Mai	1964	Chiang Mai, N Thailand†	Human	III	U70393
P19Br	1982	Chiang Mai, N Thailand	Human brain	I	U70416
KPPO34-35CT	1982	Khon Khen, NE Thailand†	Mosquito†	III	U03693
B1065	1983	South Thailand	Pig	II	U70388
B2239	1984	Chiang Mai, N Thailand	Pig blood	I	U70391
ThCMAr4492	1992	Chiang Mai, N Thailand	Mosquito†	I	D45362
JE_CM_1196	2005	Chiang Mai, N Thailand	Pig	I	DQ238602
JE_KK_80	2004	Khon Khen, NE Thailand	Pig	I	DQ111784
JE_KK_82	2004	Khon Khen, NE Thailand	Pig	I	DQ111785
JE_KK_83	2004	Khon Khen, NE Thailand	Pig	I	DQ111787
JE_KK_87	2004	Khon Khen, NE Thailand	Pig	I	DQ111788
JE_KK_577	2005	Khon Khen, NE Thailand	Pig	I	DQ238601
JE_KK_580	2005	Khon Khen, NE Thailand	Pig	I	DQ238600
JE_KK_1116	2005	Khon Khen, NE Thailand	Pig	I	DQ343290
JE_RT_36	2003	Ratchaburi, Central plain Thailand	Mosquito‡	I	DQ087975
JE_CP_49	2004	Chumphon, S Thailand	Pig	I	DQ087974
JE_CP_67	2004	Chumphon, S Thailand	Pig	I	DQ087972
JE_PK52	2004	Phuket, S Thailand	Mosquito§	I	DQ084229
VN118	1979	Vietnam	Mosquito†	III	U70420
02VN22	2002	Vietnam	Pig blood	I	AY376465
Murray Valley E.1-51	1951	Australia	Human		AF161266

*N, northern; NE, northeastern; S, southern. †Unidentified species. ‡*Culex tritaeniorhynchus.* §*Cx. quinquefasciatus*.

Twelve JEV strains were isolated, 3 from mosquitoes in 2003 and 10 from pigs in 2004 and 2005. The new JEV sequences were analyzed with a group of 22 previously published JEV strain sequences, including 6 from Thailand and 16 from other Asian countries. A phylogenetic tree was generated, and all 12 JEV new isolates fit into the same GI cluster, as did 3 other Thai strains previously isolated in 1982, 1984, and 1992 (Figure 2). Eleven of the newly identified isolates formed a subcluster (Figure 2, GIa) with 2 other strains previously isolated from Chiang Mai and Khon Ken in 1984 and 1982, respectively (B2239NThailand, P19Br NThailand); the remaining new isolate (JE KK 1116NEThailand2005) was associated with another subcluster (Figure 2, GIb), including strains isolated in 1992 from Chiang Mai (ThCMAr4492) and 3 others isolated from Vietnam, Japan, and Korea (O2VN22; JaNAr0102; and K94P05). Both subclusters were supported by 1,000 bootstrap replications and were consistent with the taxa distance (data not shown) showing introductions of GI in 1982 (within the GIa subcluster followed by a recent dispersion all over the country), and in 1992 for the GIb subcluster followed by local transmission.

GI strains appeared to cluster phylogenetically but not geographically, which suggests virus strains were transported over noncontiguous domains at variable geographic distances. Major environmental changes have occurred since the early 1950s with the increase in local and international transportation systems. Some researchers (*10*) consider the increase of the virus incidence in the human population to be associated with increased commercial activity. However, because of the low level of viremia in humans, traditionally considered dead-end hosts for JEV, it is more likely that the virus was spread within the country and to neighboring countries by migratory birds, infected domestic pigs, or infected mosquitoes (or their eggs) (*11*,*12*).

Although GIII strains were historically reported to circulate mostly in northern Thailand in the early 1980s, GI and GIII were found co-circulating from the north to the south; thereafter, only the GI strain was isolated in Thailand (*13*). The same genotype shift of GIII to GI, dating back to the early 1990s, was reported by several other Asian countries, including Japan and Korea in 1991 and Vietnam in 2001 (*14*); a steady emergence and dispersion of GI was also noticed in China in 1979, in Taiwan in the 1980s (*13*), and in Australia in 2000 (*2*). Altogether, such unique endemic expansion of GI occurred over a 25-year period in several countries of Southeast Asia, replacing the GIII genotype, which was present all over the region since the beginning of the virus genotype identification (prospectively and retrospectively).

## Conclusions

In Thailand, the epidemiologic pattern of Japanese encephalitis first showed a visible decline in incidence with the development of immunization programs, but this decline also corresponded to the late 1980s when the practice of raising pigs in the backyard evolved into industrialized pig farming and the high rate of piglet seroconversion showed an intense virus circulation. The dramatic increase of industrial pig farming and trading must have played a major role in the dispersion of JEV genotypes within past decades in Asia. Concurrently with pig farming, the culicid main vectors have changed (*14*) and such factors as their ecology, trophic preferences, host competence, and virus fitness could play a role in an evolving rural environment. Moreover, further studies are needed to clarify the expansion of JEV GI strains, including the efficiency of a human and pig GIII-derived vaccine and the role of potential cross-immunity between another circulating flavivirus (*13*).
